# Ordovician ash geochemistry and the establishment of land plants

**DOI:** 10.1186/1467-4866-13-7

**Published:** 2012-08-28

**Authors:** John Parnell, Sorcha Foster

**Affiliations:** 1 School of Geosciences, University of Aberdeen, Aberdeen, AB24 3UE, UK; 2 Victoria College, Cranmore Park, Belfast, BT9 6JA, UK

**Keywords:** Ash geochemistry, Tuff, Land plants, Chemical index of alteration, Phosphorus, Biomass, Ordovician

## Abstract

The colonization of the terrestrial environment by land plants transformed the planetary surface and its biota, and shifted the balance of Earth’s biomass from the subsurface towards the surface. However there was a long delay between the formation of palaeosols (soils) on the land surface and the key stage of plant colonization. The record of palaeosols, and their colonization by fungi and lichens extends well back into the Precambrian. While these early soils provided a potential substrate, they were generally leached of nutrients as part of the weathering process. In contrast, volcanic ash falls provide a geochemically favourable substrate that is both nutrient-rich and has high water retention, making them good hosts to land plants. An anomalously extensive system of volcanic arcs generated unprecedented volumes of lava and volcanic ash (tuff) during the Ordovician. The earliest, mid-Ordovician, records of plant spores coincide with these widespread volcanic deposits, suggesting the possibility of a genetic relationship. The ash constituted a global environment of nutrient-laden, water-saturated soil that could be exploited to maximum advantage by the evolving anchoring systems of land plants. The rapid and pervasive inoculation of modern volcanic ash by plant spores, and symbiotic nitrogen-fixing fungi, suggests that the Ordovician ash must have received a substantial load of the earliest spores and their chemistry favoured plant development. In particular, high phosphorus levels in ash were favourable to plant growth. This may have allowed photosynthesizers to diversify and enlarge, and transform the surface of the planet.

## Background

The establishment of land plants in the terrestrial environment brought about a fundamental transformation of the Earth’s surface [1-7]. It involved new soils and soil microbiota, greatly enhanced biological weathering and new controls on landforms and erosion, a new food chain, and new habitats for animals that increased their diversity. It also enhanced the influence of photosynthesizers on the planet's atmosphere, increasing oxygen concentrations and drawing down carbon dioxide by biological weathering [1]. The Earth’s surface biomass is now dominated by land plants [8], and is so extensive that the occurrence of life on Earth would be evident to observers from space [9]. The establishment of this high surface biomass represented a crucial shift from a planet dominated by subsurface life to one in which surface life became proportionately significant. This change intrinsically involved an increase in the proportion of life ultimately supported by photosynthesis (carbon dioxide) rather than hydrogen.

Understanding the colonization of the land surface by plants requires us to identify if special geochemical circumstances had arisen to promote it, or if it was simply an aspect of a wider diversification of life into new niches. This event is dated to the early/mid-Ordovician. There are several records of Ordovician plant spores, extending back to an Arenig (Floian; 476 Ma) occurrence of liverwort-type spores [10-13], and fungal hyphae in the Ordovician could have been closely associated with evolving plants [14,15]. Plant growth is envisaged to have been sufficiently extensive and well-anchored to trigger the end-Ordovician glaciation by weathering-drawdown of CO_2_[7]. An essential requirement to allow colonization of the subaerial environment was the availability of nutrients in the soil rather than through water. The ready availability of nutrients requires some kind of soil, in which mineral matter can dissolve into pore waters at a fast rate. For much of Earth’s history, the land surface was bare rock or thin microbial crust [2], and soils were weathering products that somehow survived the fast rate of erosion possible when there were no stabilizing land plants. Occasionally, volcanic ash fall-out contributed to the surface detritus. However, tuff (lithified volcanic ash) formation was particularly sustained and widespread in the Ordovician. We show here that the chemistry of Ordovician tuffs indicates their potential role in supplying nutrients to the earliest land plants. Data sets (see Additional file 1 for detailed data and sources) were chosen based upon the availability of multiple measurements, data required to calculate CIA (chemical alteration index) values, details of analytical methods, and lack of high-grade metamorphism. CIA values are given as the ratio Al_2_O_3_/(Al_2_O_3_ + CaO + Na_2_O + K_2_O) [16].

## Results and discussion

### Ordovician volcanic activity

**Figure 1:**
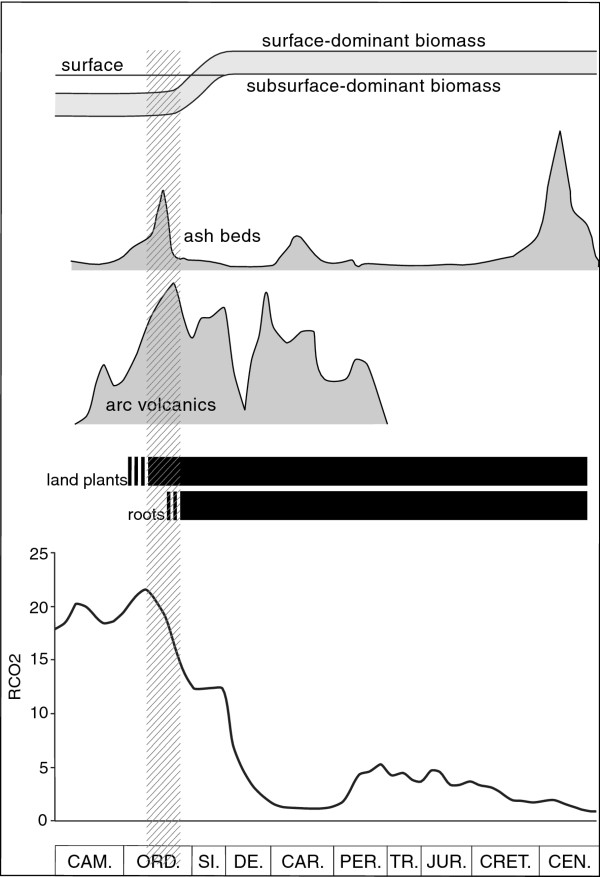
**Intensity of volcanic activity through the Phanerozoic.** Activity represented by relative changes in volcanic arc volume [17], and frequency of ash bed deposition [18]. Ordovician peak in intensity is coincident with earliest records of plants [11,12], and decline in atmospheric CO_2_ (RCO_2_ is concentration compared to present atmosphere: [1]). Consequence is a shift of biomass from subsurface to surface. True roots are known from the Siluro-Devonian, but an earlier soil anchoring system is possible [52].

**Figure 2:**
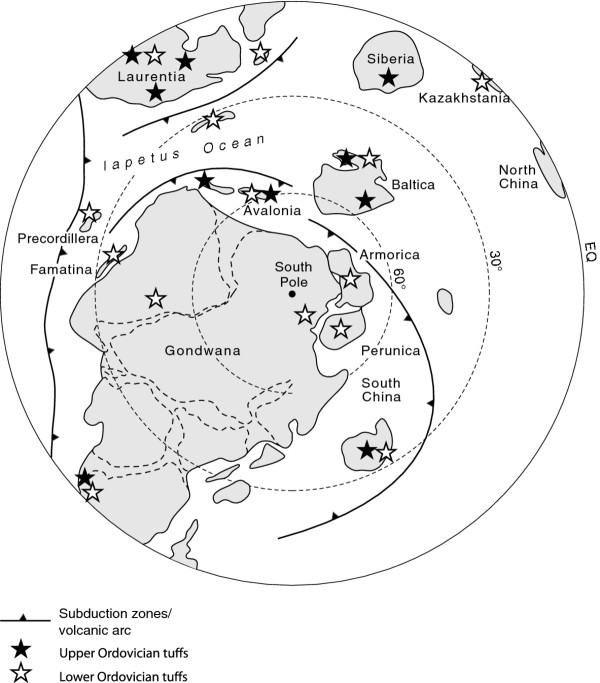
**Palaeogeographic reconstruction of the Southern Hemisphere at about 470 Ma.** Ordovician tuffs recorded on all major continents, and especially in vicinity of volcanic arcs. Map is modified from [61]; data sources in Additional file 1.

The quantification of volcanic activity on a global scale is difficult in deep geological time, but two databases assembled as proxies for global volcanic activity, based on island arc volcanism [17], and numbers of ash beds [18] both highlight the Ordovician as a period of anomalous volcanism (Figure 1). This was one of the most intense periods of volcanic activity in the Phanerozoic, and the first intense period following the ‘explosion’ of life at about the Precambrian-Cambrian boundary. This activity has been attributed to the formation of a superplume [19], accelerated sea floor spreading [20], and global reorganization of plates following the assembly of Gondwana [21]. An abundance of ophiolites containing Ordovician volcanic rocks has allowed the mapping of the Ordovician system of subduction zones and related volcanic arcs (Figure 2). There were multiple volcanic arcs, like the West Pacific today, in several parts of the globe, including both margins of the Iapetus Ocean, central Asia and the Andean margin of Gondwana (Figure 2), and they had great strike-length [21]. The extensive arcs are associated with anomalous deposits of volcanic ash (lithified as tuff, bentonite). Large volumes of ash are a product of explosive volcanism that is typical of periods of accelerated subduction, as at present, and especially in the Ordovician [18,22,23]. Ash from volcanic arcs was carried by winds hundreds to thousands of kilometres into continental interiors [24].

Tuffs and basaltic lavas occur through the entire Ordovician and into the Silurian, but are particularly widespread in the Arenig-Llanvirn and Caradoc (Floian-Dapingian and Sandbian respectively) [25]. The Caradoc tuffs in particular occurred on a huge scale, correlated across the Iapetus Ocean from Laurentia to Baltica (Figure 2), and reaching a volume of over a thousand cubic kilometres [22], more than an order of magnitude greater than from the Krakatoa eruption of 1883, the greatest known eruption of historical times. The mere survival of macroscopic tuff beds suggests exceptionally large eruptions, as otherwise the ash would be mixed into the background sediment [18,22,23]. Much ash would have been deposited in the ocean, now largely lost from the geological record by subduction. However, much also fell in shallow water and terrestrial environments, hence their widespread preservation. The exposure of Ordovician volcanic rocks to contemporary subaerial weathering is evidenced by reddened basalts and even palaeosols on basalts [26]. Extensive weathering of these volcanic rocks is also implicated in global isotopic and climatic signals [27,28].

### Volcanic ash chemistry and plant growth

The value of volcanic ash to plant life is implied by the fact that the most densely populated area of the world in Indonesia, and other high-density populations in Africa, are on young volcanic ash with very high soil productivity [29], the use of crushed tuffs as fertilizers [30,31], and the rapid recovery of plants on ash-covered terrains following volcanic eruptions [29]. In even the short time elapsed since the outpouring of troublesome ash from Eyjafjallajökull, Iceland in 2010, there have been several reports from Icelandic farmers describing increased plant yields and the deliberate use of the ash as a fertilizer [32,33]. The fertilizing potential of volcanic ash is also evident in its effects on phytoplankton in the oceans [34,35]. Volcanic ashes, and associated basaltic lavas, are relatively rich in the nutrients required by plants, including iron, calcium, potassium, magnesium, sulphur, nitrogen and phosphorus. However the two elements most likely to be limiting are phosphorus and nitrogen [29,36].

**Figure 3:**
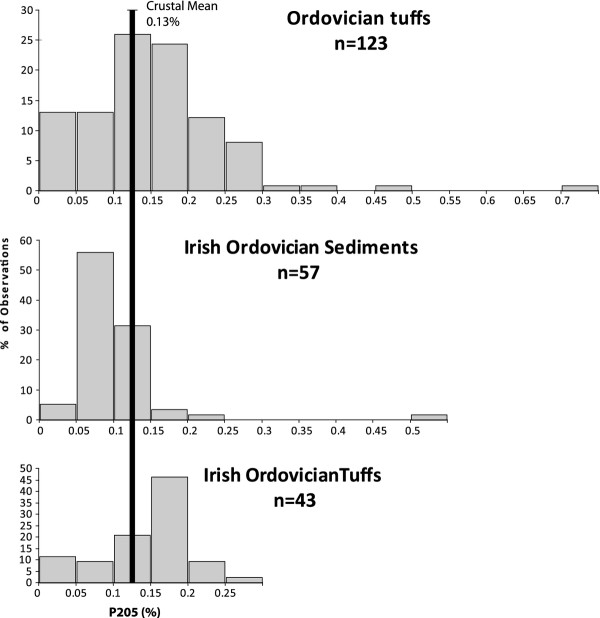
**Phosphorus contents (wt.% P**_**2**_**O**_**5**_**) for Ordovician tuffs.** Most values exceed mean crustal value of 0.13%. Data set for Ireland [40] shows higher values in Ordovician tuffs compared to contemporaneous sediments. Detailed data and sources in Additional file 1.

Phosphorus concentrations in modern ashes include 0.15% and 0.17% P_2_O_5_ in ashes from Japan and the Philippines respectively, both above the crustal mean of 0.13%, and both adequate for plant growth [37]. Experiments using volcanic ash as a plant growth substrate have demonstrated its importance as a source of phosphorus [38]. Data from Ordovician tuffs show that the majority have P_2_O_5_ contents greater than the crustal mean (Figure 3), and that in mixed volcanic-sedimentary successions, tuffs are more phosphorus-rich than the normal sediments [39,40]. Considering that these values may be depleted from original concentrations by leaching, they indicate that the Ordovician ashes contained adequate phosphorus for plant growth. The phosphorus in these rocks is, like today, mainly in the form of apatite. Apatite is relatively soluble in (acidic) rain water, so can be readily liberated from ash into soil solutions [37]. The survival of much apatite in Ordovician tuffs today shows that they had potential for long-term release of phosphorus.

Nitrogen availability is less easy to quantify in ancient systems, as most nitrogen is introduced to ashes by rain water or by fixation from symbiotic micro-organisms [37]. Nevertheless, there is evidence that microbial communities were colonizing soils before the Ordovician, in the Precambrian [2,41-43] and nitrogen fixation was already comparable to the present day, including in terrestrial systems [44]. More specifically, the land surface was probably colonized already by fungi and lichens [42,43,45], and they and the primitive liverwort plants established in the Ordovician [10] are all capable of nitrogen fixation. Mycorrhizal fungi are believed to have been instrumental to the colonization of land by plants by symbiotic nutrient acquisition [46], in a relationship established by Ordovician times [14,15,45,47]. These fungi inoculate volcanic ash by wind following modern eruptions [48]. Nitrogen fixation might be enhanced by volcanic lightning in ash clouds [49,50], and nitrogen delivery would also be possible through weathering of the ashes. There was, therefore, strong potential for nitrogen availability in ash to the earliest plants.

The physical attributes of volcanic ash allow good drainage, but also high retention of plant-available water [37,51], which would have been essential to the earliest plants occupying moist lowland areas. It also provides excellent ‘tilth’, the physical condition related to fitness as a seed-bed [37]. The combination of water and nutrient availability would have made ashes a favourable setting for the seeding of primitive plants. Although the oldest true roots recorded to date are of Silurian age, some form of soil anchoring system which absorbed nutrients is probable from the earliest stages of land colonization [52,53]. The permeable but water-retaining soils that support plants widely today would not form until plant roots themselves had evolved to secrete rock-consuming organic acids and stabilize the residual grains.

The importance of volcanic ash, via plants, to the whole food chain, is exemplified today in an extensively studied ecosystem in the Serengeti, Tanzania. High phosphorus levels in the ash are conferred to the grassland vegetation, which is consumed by wildebeests, who are eaten by animal predators and whose dung supports huge numbers of insects [54,55].

### Volcanic ash compared to earlier soils

**Figure 4:**
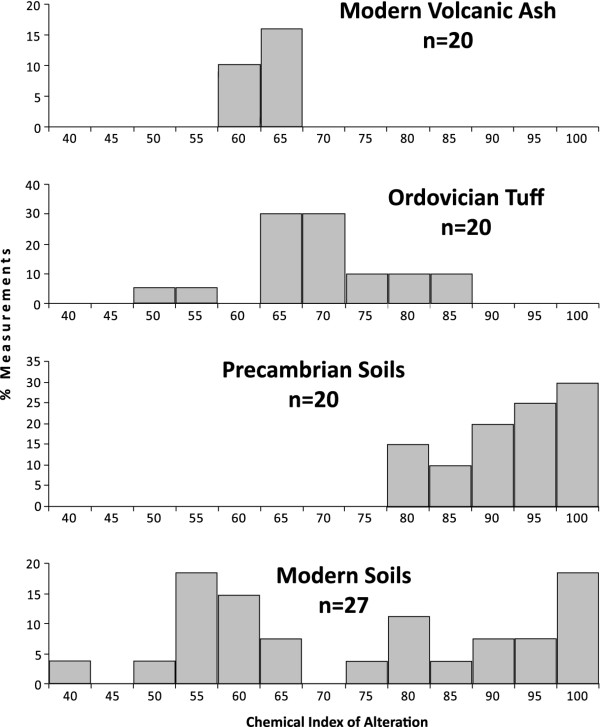
**Chemical Index of Alteration (CIA) for ashes and soils.** Modern [60] and Ordovician volcanic ash (tuff) (sources in Additional file 1) show lower values than Precambrian soils [56]. Modern soils [56] show wide-ranging values, reflecting varying involvement of plants in different climates.

The geochemical contrast between earlier, Precambrian, soils and the Ordovician volcanic ashes is evident in values for CIA (Chemical Index of Alteration). In Precambrian soils, alkalis (K, Na, Ca), and other, additional, elements not used to calculate the alteration index, were typically all leached to leave high CIA values of 75 to 100 [56] (Figure 4). In Ordovician tuffs this depletion is not observed, and even allowing that some tuffs may be diagenetically enriched in potassium [24], there was clearly a greater nutrient retention in the ashes (Figure 4). The significance of the lower CIA values (mostly 60–70) for the Ordovician tuffs is emphasized by higher values (70–80) for Ordovician siliciclastic deposits in several parts of the world [57-59]. The tuffs were a particularly fertile protolith. Similarly, modern ashes have relatively low CIA values [60] (Figure 4). Given that tuff beds were deposited almost instantaneously, they represent a flux of phosphorus and other nutrients to the surface greatly in excess of that during normal sedimentation or soil formation. Progressive exposure and erosion of the tuffs could have delivered nutrient-rich material available to plants at the surface over a prolonged time.

## Conclusion

There is clearly a coincidence between the earliest records of plants and the timing of exceptional volcanic activity and ash-fall. Ash was not essential to plant growth, but was widely available, and must have received a substantial load of the available spores. Where tuffs survived subaerial erosion, they could have remained at the land surface for prolonged periods, as found in highly populated regions today [29], so that repeated inoculation by spores was unavoidable. Given its beneficial chemical and physical properties, spores embedded in ash must have had a relatively favourable chance of germination. The strong suitability of these volcanic deposits to support plant growth suggests that they deserve detailed scrutiny for early plant fossils. The compaction of highly porous ashes during lithification to tuffs will make such evidence difficult to discern, and the well-drained environment of ashes is not conducive to fossil preservation. However, rare palaeosols formed on Ordovician basalts show mineral alteration attributed to nonvascular plants [26], providing evidence that volcanic rocks may indeed have been suitable substrates and thereby played an important role in the colonization of land by plants.

## Competing interests

Both authors declare that they have no competing interests.

## Authors’ contributions

JP initiated and directed the project, and put the data in geological context. SF assembled and processed the geochemical data and ash distribution data. Both authors read and approved the final manuscript.

## Supplementary Material

Additional file 1 **Ordovician ash geochemistry and the establishment of land plant [**[62-84]**].** Click here for file
