# Supplementary material for: Ordovician ash geochemistry and the establishment of land plants
Source: Geochem Trans. 2012 Aug 28;13:7. doi: 10.1186/1467-4866-13-7 (PMC3485180; doi:10.1186/1467-4866-13-7)
Supplement: Additional file 1 — Ordovician ash geochemistry and the establishment of land plant [[62-84]]. [file 1467-4866-13-7-S1.docx]

**Ordovician ash geochemistry and the establishment of land plants**

**Additional data files**

Data sources for figures

Fig. 2: Tuff distribution

Dronov, A.V., Huff, W.D., Kanygin, A.V. & Gonta, T.V. K-bentonites in the Upper Ordovician of the Siberian Platform. In: Gutiérrez-Marco, J.C. et al. (eds) Ordovician of the World. Instituto Geológico y Minero de España, Madrid, 135-141 (2011).

Gutiérrez-Alonso, G., Fernández-Suárez, Gutiérrez-Marco, J.C., Corfu, F., Murphy, J.B. & Suárez, M. U-Pb depositional age for the upper Barrios Formation (Armorican Quartzite facies) in the Cantabrian Zone of Iberia: Implications for stratigraphic correlation and paleogeography. Geol. Soc. Amer. Spec. Paper 423, 287-296 (2007).

Haynes, J.T., Huff, W.D. & Melson, W.G. Major Ordovician tephras generated by caldera-forming explosive volcanism on continental crust: Evidence from biotite compositions. In: Gutiérrez-Marco, J.C. et al. (eds) Ordovician of the World. Instituto Geológico y Minero de España, Madrid, 229-235 (2011).

Huff, W.D. & Bergstrom, S.M. Castlemainian K-bentonite beds in the Ningkuo Formation of the Jiangshan Province – The first Lower Ordovician K-bentonites found in China. Palaeoworld, 5, 101-105 (1995).

Huff, W.D., Bergstrom, S.M., Kolata, D.R., Cingolani, C.A. & Astini, R.A. Ordovician K-bentonites in the Argentine Precordillera: relations to Gondwana margin evolution. Geological Society, London, Special Publications, 142, 107-126 (1998).

MacLachlan, K., O’Brien, B.H. & Dunning, G.R. Redefinition of the Wild Bight Group, Newfoundland: implications for models of island-arc evolution in the Exploits Subzone. Canadian Journal of Earth Science, 38, 889-907 (2001).

Popov, L.E., Bassett, M.G., Zhemchuzhnikov, V.G., Holmer, L.E. & Klishevich, I.A. Gondwanan faunal signatures from Early Palaeozoic terranes of Kazakhstan and Central Asia: evidence and tectonic implications. Geological Society, London, Special Publications, 325, 23-64 (2009).

Ramos, E., Marzo, M., de Gibert, J.M., Tawengi, K.S., Khoja, A.A. & Bolatti, N.D. Stratigraphy and sedimentology of the Middle Ordovician Hawaz Formation (Murzuq Basin, Libya). AAPG Bull. 90, 1309-1336 (2006).

Ross, R.J. The Ordovician system: Progress and Problems. Annual Reviews of Earth and Planetary Science, 12, 307-335 (1984).

Ryan, K.M. & Williams, D.M. Testing the reliability of discrimination diagrams for determining the tectonic depositional environment of ancient sedimentary basins. Chemical Geology, 242, 103-125 (2007).

Sliaupa, S. Ordovician-Silurian metabentonites in the Baltic Basin: A record of surrounding Caledonian volcanic activity. Geophysical Journal, 22, 128-129 (2000).

Tait, J., Bachtadse, H. & Soffel, H. New palaeomagnetic constraints on the position of central Bohemia during Early Ordovician times. Geophys. J. Int. 116, 131-140 (1994).

Villas, E., Gisbert, J. & Montesinos, R. Brachiopods from volcaniclastic middle and upper Ordovician of Asturias (Northern Spain). J. Paleont. 63, 554-565 (1989).

Figs. 3, 4: Tuff chemistry

Álvaro, J.J., Ezzouhairi, H., Ribeiro, M.L., Ramos, J.F. & Solá, A.R. Early Ordovician volcanism in the Iberian Chains (NE Spain) and its influence on the preservation of shell concentrations. Bulletin de la Societe Géologique de France, 179, 569-581 (2008).

Bahlburg, H., Carlotto, V. & Cardenas, J. Evidence of Early to Middle Ordovician arc volcanism in the Cordillera Oriental and Altiplano of southern Peru, Ollantaytambo Formation and Umachiri beds. Journal of South American Earth Sciences, 22, 52-65 (2006).

Beddoe-Stephens, B., Petterson, M.G., Millward, D. & Marriner, G.F. Geochemical variation and magmatic cyclicity within an Ordovician continental-arc volcanic field: the lower Borrowdale Volcanic Group, English Lake District. Journal of Volcanology and Geothermal Research, 65, 81-110 (1995).

Brusewitz, A.M. Chemical and physical properties of Paleozoic potassium bentonites from Kinnekulle, Sweden. Clay and Clay Minerals, 34, 442-454 (1986).

Delano, J.W., Schirnick, C., Bock, B., Kidd, W.S.F., Heizler, M.T., Putman, G.W., De Long, S.E. & Ohr, M. Petrology and geochemistry of Ordovician K-bentonites in New York State: Constraints on the nature of a volcanic arc. Journal of Geology, 98, 157-170 (1990).

Fritz, W.J. & Stillman, C.J. A subaqueous welded tuff from the Ordovician of County Waterford, Ireland. Journal of Volcanology and Geothermal Research, 70, 91-106 (1996).

Fritz, W.J. & Vanko, D.A. Geochemistry and origin of a black mudstone in a volcaniclastic environment, Ordovician Lower Rhyolitic Tuff Formation, North Wales, UK. Sedimentology, 39, 663-674 (1992).

Huff, W.D., Merriman, R.J., Morgan, D.J. & Roberts, B. Distribution and tectonic setting of Ordovician K-bentonites in the United Kingdom. Geological Magazine, 130, 93-100 (1993).

Leat, P.T. & Thorpe, R.S. Ordovician volcanism in the Welsh Borderland, 123, 629-640 (1986).

Leo, G.W. Trondhjeimite and metamorphosed quartz keratophyre tuff of the Ammonoosuc Volcanics (Ordovician), western New Hampshire and adjacent Vermont and Massachusetts. Geological Society of America Bulletin, 96, 1493-1507 (1985).

Orton, G. Geochemical correlation of Ordovician flow tuffs in North Wales. Geological Journal, 27, 317-338 (1992).

Wilson, R.A. Geochemistry and petrogenesis of Ordovician arc-related mafic volcanic rocks in the Popelogan Inlier, northern New Brunswick. Canadian Journal of Earth Science, 40, 1171-1189 (2003).

Young, T.P., Gibbons, W. & McCarroll, D. Geology of the Country around Pwllheli. Memoir, British Geological Survey. London, The Stationery Office (2002).

CIA data for Ordovician tuffs

| **Country** | **Formation** | **Age** | **CIA** | **Reference** | **n** |
| --- | --- | --- | --- | --- | --- |
| Scotland | Hartfell Shale | Caradoc-Ashgill | 73.9 | Huff et al. 1993 | 3 |
| Canada | Goulette Brook Formation | Caradoc | 49.95 | Wilson 2003 | 8 |
| England | Borrowdale Volcanic Group | Caradoc | 62.31 | Beddoe-Stephens et al. 1994 | 4 |
| England | Hagley Volcanic Group | Caradoc | 67.11 | Leat & Thorpe 1985 | 2 |
| Ireland | Metal Man Tuff | Caradoc | 66.75 | Fritz & Stillman 1995 | 12 |
| Sweeden | Kinnekulle Bentonites | Caradoc | 79.13 | Brusewitz 1986 | 17 |
| USA | Ammonoosuc Keratophyre | Caradoc | 63.3 | Leo 1985 | 8 |
| USA | Utica Shale | Caradoc | 82.22 | Delano et al 1990 | 1 |
| Wales | Allt Fawr Rhyolitic Tuff | Caradoc | 66.86 | Young et al. 2002 | 3 |
| Wales | Lower Rhyolitic Tuff | Caradoc | 54 | Fritz & Vanko 1992 | 2 |
| Wales | Racks and Garth Tuff | Caradoc | 67.93 | Orton 1992 | 13 |
| Wales | Trygarn Formation | Llanvirn | 66.48 | Young et al. 2002 | 2 |
| Peru | Umachiri Beds | Arenig-Llanvirn | 82.46 | Balburg et al 2006 | 8 |
| Ireland | Derrylea Formation | Arenig | 67.19 | Ryan & Williams 2007 | 6 |
| Ireland | Mweelrea Formation | Arenig | 62.59 | Ryan & Williams 2007 | 19 |
| Ireland | Rosroe Formation | Arenig | 61.85 | Ryan & Williams 2007 | 8 |
| Ireland | Sheeffry Formation | Arenig | 63.29 | Ryan & Williams 2007 | 11 |
| Peru | Ollantaytambo Formation | Arenig | 65.05 | Balburg et al 2006 | 13 |
| Spain | Bubeirca Tuff | Tremadoc | 73.7 | Alvaro et al. 2008 | 2 |
| Spain | Tranquera Tuff | Tremadoc | 75.58 | Alvaro et al. 2008 | 2 |
